# Zanubrutinib-induced liver injury: a case report and literature review

**DOI:** 10.1186/s12876-021-01825-2

**Published:** 2021-05-29

**Authors:** Edmond Atallah, Pramudi Wijayasiri, Nicole Cianci, Khorrum Abdullah, Abhik Mukherjee, Guruprasad P. Aithal

**Affiliations:** 1 Nottingham Digestive Diseases Centre, School of Medicine, University of Nottingham, Nottingham, UK; 2 National Institute for Health Research (NIHR) Nottingham Biomedical Research Centre, Nottingham University Hospitals NHS Trust and the University of Nottingham, Nottingham, UK; 3 University Hospitals Birmingham (UHB), Birmingham, UK; 4 Histopathology, Nottingham University Hospitals NHS Trust, Nottingham, UK; 5 Division of Cancer and Stem Cells, School of Medicine, University of Nottingham, Nottingham, UK

**Keywords:** Zanubrutinib, hepatotoxicity, Drug-induced liver injury, Case report, RUCAM

## Abstract

**Background:**

Zanubrutinib is a Bruton’s tyrosine kinase inhibitor that has been recently licensed in refractory mantle cell lymphoma and under assessment in phase 3 clinical trials for other B cell malignancies. To date, there are no reported cases of hepatotoxicity secondary to zanubrutinib. We report the first case of severe liver injury due to zanubrutinib.

**Case presentation:**

A 56-year-old Caucasian male with a history of relapsed lymphoplasmacytic lymphoma was admitted to the hospital with new-onset jaundice, choluria, and pruritus for 10 days. He had been on zanubrutinib as part of a clinical trial for 30 months. His blood profile showed a severe hepatocellular injury with jaundice (alanine transaminase 2474 IU/L and total bilirubin 141 umol/L with mild coagulopathy). He had an extensive work-up including virology, autoimmune, and metabolic profiles in addition to abdominal ultrasound with no alternative explanation found for his liver injury. Zanubrutinib-induced liver injury was suspected, and causality assessment by the updated Roussel Uclaf Causality Assessment Method score showed a probable causal relationship with zanubrutinib. His liver histology was also consistent with drug-induced liver injury. His liver biochemistry improved following cessation of zanubrutinib and normalised after 8 weeks.

**Conclusion:**

We report the first case of severe liver injury secondary to zanubrutinib after 30 months of treatment. This case raises clinical awareness regarding zanubrutinib-induced liver toxicity and the importance of drug withdrawal in the event of liver injury.

## Background

Idiosyncratic drug-induced liver injury (DILI) is an important cause of acute liver failure in Europe and the United States (US) [1, 2] and is one of the most common reasons for drug withdrawal from the pharmaceutical market [3, 4]. These implications and its idiosyncratic nature pose a particular challenge both to clinicians and the pharmaceutical industry, who must be vigilant for liver-related adverse effects in new drugs. The type of liver injury is classified as hepatocellular, cholestatic, or mixed depending on the pattern of liver profile at the time of injury. Establishing a temporal relationship is crucial in making the diagnosis of DILI and, the updated Roussel Uclaf Causality Assessment Method (RUCAM) is the most common diagnostic tool used in clinical practice and trials for DILI definitions and inclusion [5, 6]. The mainstay of management of DILI is establishing the diagnosis and stopping the offending drug, which often results in the resolution of liver injury. However, in severe cases, DILI may progress to acute liver failure, requiring liver transplantation or leading to death [5].

Waldenström’s macroglobulinaemia (WM) is a chronic, indolent, B-cell lymphoproliferative disorder characterised by bone marrow infiltration with lymphoplasmacytic cells that secrete monoclonal immunoglobulin M (IgM) and activate the B-cell receptor signalling complex, of which Bruton tyrosine kinase (BTK) is a crucial enzyme [7, 8]. BTK inhibitors have recently emerged as an effective treatment option for relapsed WM. Zanubrutinib is a novel potent BTK inhibitor that has shown a good safety profile in clinical studies [9]. We report the first case of severe liver injury following zanubrutinib therapy with a literature review of BTK inhibitors-induced liver injury.

## Case presentation

The case involved a 56-year-old Caucasian man who was diagnosed with Waldenström’s macroglobulinaemia at the age of 35 and previously treated with fludarabine and cyclophosphomide chemotherapy and plasma exchange. His past medical history also included mild psoriasis and being overweight (BMI 30). He did not have any regular medication. He provided a history of drinking 300 g of alcohol a week and had been a lifelong smoker of 10 cigarettes per day. Following a 10-year monitoring period when he was asymptomatic, his paraprotein levels increased significantly, and he had widespread lymphadenopathy on cross-sectional imaging. His bone marrow biopsy showed 20–30 % infiltration with low-grade lymphoplasmacytic lymphoma and MYD88 disease with CXCR4 wild type.

He subsequently participated in phase 3 BeiGene randomised clinical trial comparing zanubrutinib with ibrutinib for relapsing WM, and he was randomised to the zanubrutinib arm. He took zanubrutinib 160 mg twice daily continuously in addition to aciclovir and co-trimoxazole as prophylaxis for opportunistic infections. His blood profile was monitored every 4 weeks, as shown in Fig. 4. After 28 months of treatment, he had an asymptomatic increase in transaminases and total bilirubin (TB), but he continued to take the drug. Following 8 weeks, he developed pale stools, dark urine, pruritis and jaundice and was admitted to the emergency department. He had no clinical signs of liver failure on examination, and his admission blood profile showed markedly elevated transaminases: ALT 2474 (upper limit of normal (ULN): 45 IU/L), AST 1257 (ULN: 35 IU/L), ALP 114 (ULN: 130 IU/L), TB 141 (ULN: 21 umol/L), PT 14 (ULN: 12 s). He was not exposed to any new medication or herbal remedies in the 6 months prior to the onset of his symptoms. Zanubrutinib was withheld as a suspected cause of liver injury, and a full liver screen was undertaken. His liver ultrasound showed a non-dilated biliary tree and a normal outline of the liver with echo-bright texture in keeping with fatty changes. His serological liver screen was negative, excluding other possible causes of liver injury; results are summarised in Table 1.

**Fig. 1:**
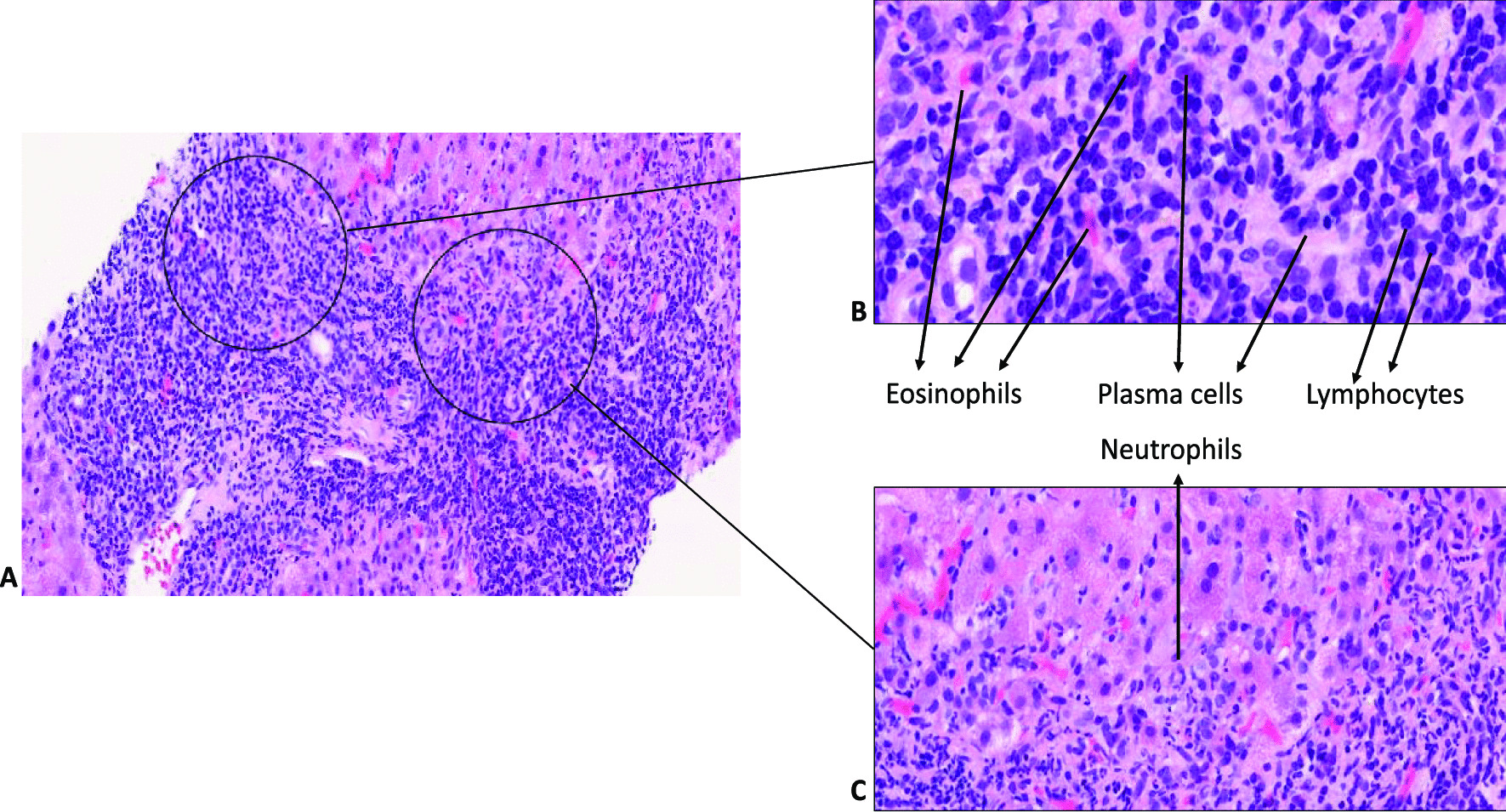
Histopathological changes of portal tracts in zanubritinib-induced liver injury. **A** Prominent portal and interface inflammation; **B** high-power imaging showing inflamed portal tracts with lymphocytes, plasma cells, neutrophils and eosinophils. **C** Inflammation at the interface with neutrophils infiltration.

**Fig. 2:**
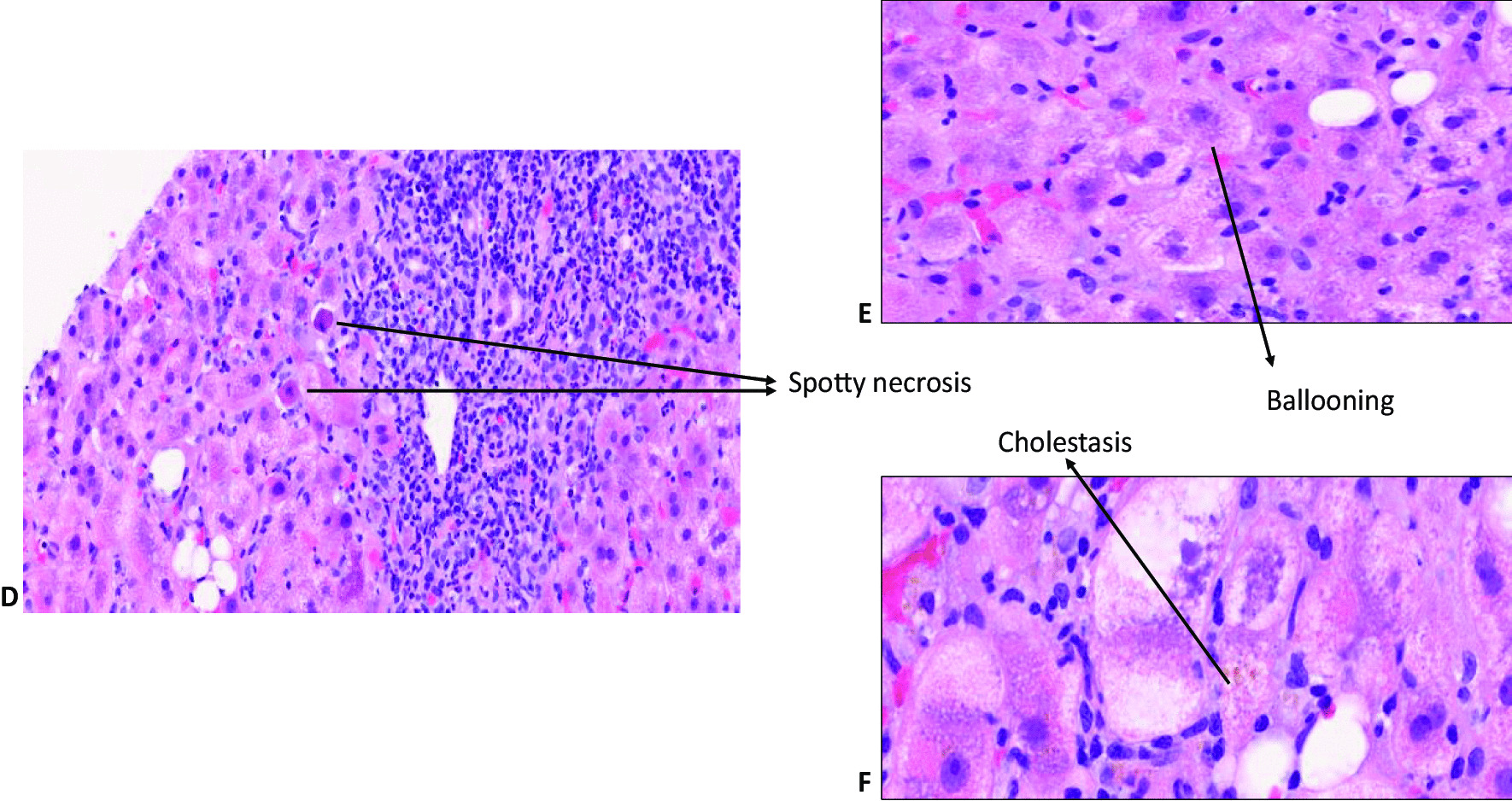
Histopathological changes of liver lobules in zanubritinib-induced liver injury: Lobular inflammation with spotty necrosis (**D**), ballooning (**E**) and cholestasis (**F**)

**Fig. 3:**
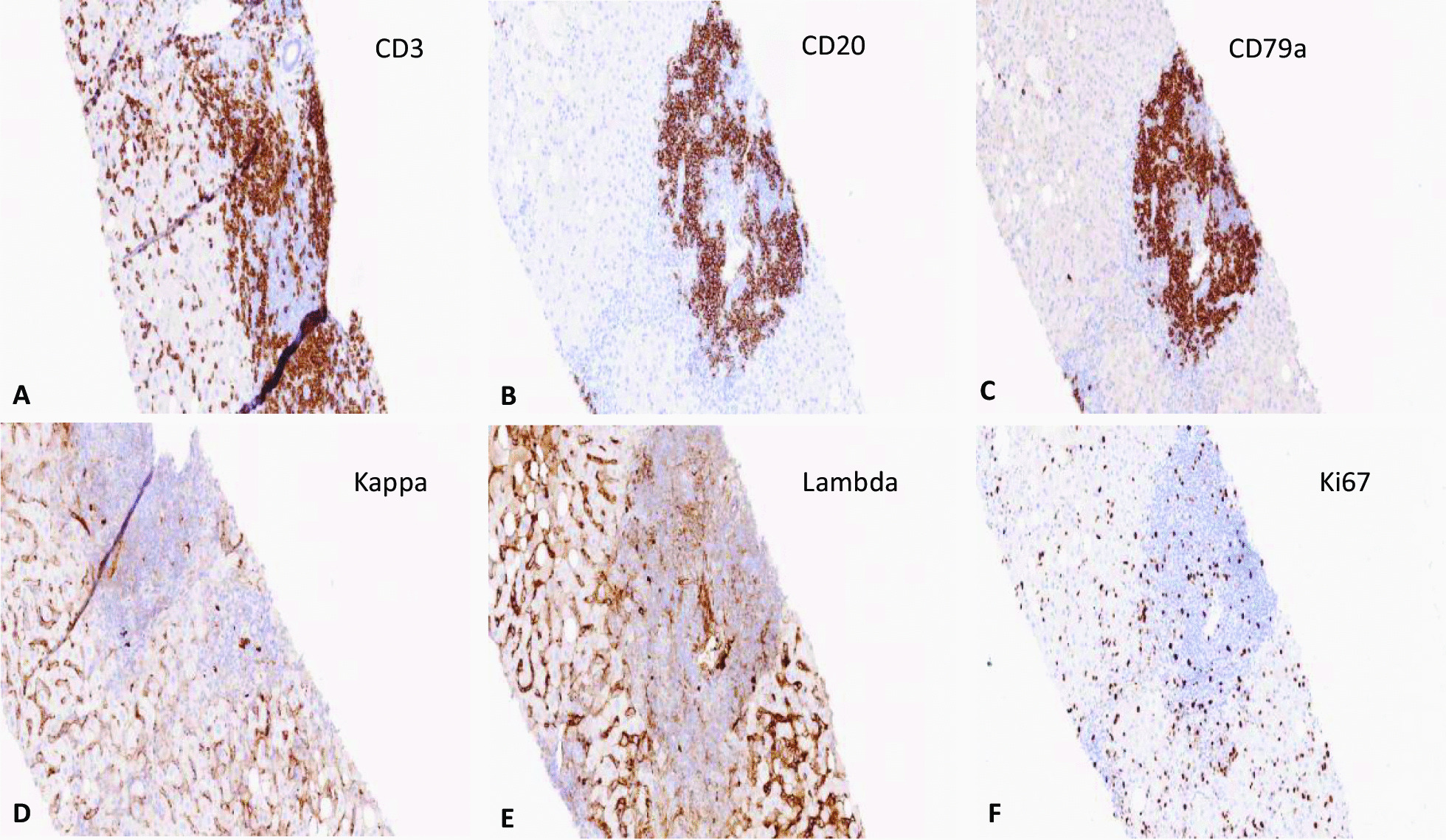
Immunohistochemical staining of lymphocytic infiltration. Most lymphoid cells within the portal tracts and sinusoids were CD3 positive T cells (**A**), with some CD20 and CD79a positive B cells present within portal tracts but not extending into the surrounding hepatic parenchyma (**B**, **C**). No evidence of light chain restriction was seen on immunohistochemical staining for kappa and lambda light chains (**D**, **E**). Ki67 index was low (**F**)

The liver injury was hepatocellular with an R-value of 63.2, and the updated RUCAM causality assessment score for zanubrutinib was 7, indicating “probable drug-induced liver injury” [6]. He undertook a liver biopsy, which showed patchy necrosis, cholestasis and moderate to severe lobular and portal inflammation suggestive of a DILI without any evidence of alcohol-related liver disease, as shown in Figs. 1 and 2. Due to his underlying lymphoplasmacytic lymphoma, portal lymphoid infiltration was further characterised by immunohistochemistry, and the features were in line with a reactive lymphoid infiltrate, with no evidence of light chain restriction, as shown in Fig. 3.

**Fig. 4:**
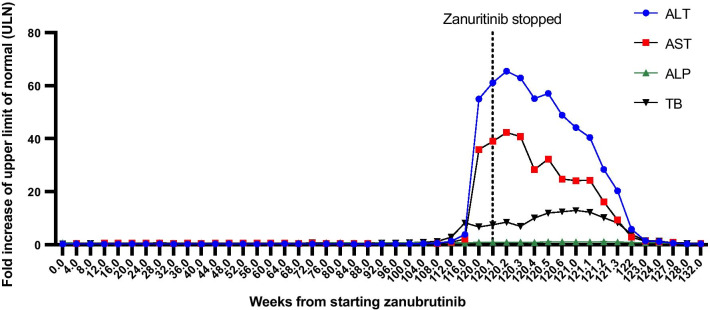
Evolution of liver biochemistry since starting zanubritinib

**Table 1:** Liver profile and serological liver screen

	Lab values	Normal range	Virology screen	
ALT (IU/L)	2474	0–45	IgM anti-HAV	Negative
AST (IU/L)	1257	0–35	HBsAg	Negative
ALP (IU/L)	114	40–130	Anti-HCV	Negative
Total Bilirubin (umol/L)	141	0–21	IgM anti-HEV	Negative
Albumin (g/L)	38	35–52	HEV RNA	Not detected
Prothrombin time (s)	14	10–12	IgM anti-CMV	Negative
Lipase (U/L)	23	0–67	CMV DNA	Not detected
a1-antitrypsin(g/L)	2.0	1.0–2.1	IgM anti-EBV	Negative
Paracetamol (mg/L)	< 10	< 10	EBV DNA	Not detected
Ferritin (ug/L)	4558	25–350	Autoimmune liver screen	
Caeruloplasmin (g/L)	0.282	0.2–0.6	ANA	Negative
IgG (mg/dL)	740	530–1650	AMA	Negative
IgA (mg/dL)	100	70–400	ASMA	Negative
IgM (mg/dL)	400	50–190	LKM-1	Negative

Patient’s liver enzymes improved following drug cessation, and he was discharged after a 9-day long admission. His liver profile normalised 28 days after discharge (Fig. 4), and his Zanubrutinib had been stopped permanently. He was started on bendamustine and rituximab due to the rise in his paraprotein levels, and his liver enzymes remained within normal range.

## Discussion and conclusion

Zanubrutinib is an orally available small-molecule inhibitor of Bruton’s tyrosine kinase (BTK). It is the third drug in this class having received approval for treatment of refractory mantle cell lymphoma after ibrutinib and acalabrutinib. It has been approved in the USA since 2019 and has been under evaluation for use in other B cell disorders. Safety data from pre-licensing clinical trials showed that zanubrutinib is generally well tolerated, with frequent but mild side effects. Although mild elevations in liver enzymes were frequent, with less than 1 % of patients developed grade 3 or 4 ALT or bilirubin elevation, no severe hepatic impairment was reported [10, 11]. Combined data drawn from over 600 patients showed that LFT abnormalities were frequent but often mild. ALT elevations arose in 28 % and bilirubin levels in 24 % of subjects but were above five times the upper limit of normal (ULN) in less than 1 %. There were no reports of clinically apparent liver injury or early discontinuation because of liver injury or liver-related deaths [10]. Moreover, phase 1 clinical trials of Zanubrutinib in patients with B cell malignancies did not report hepatotoxicity or ALT elevation [12, 13].

To compare liver toxicity from zanubritinib with other BTK inhibitors, we reviewed the literature and summarised the available evidence from case reports of BTK-inhibitors-induced liver injury published to date in Table 2. Ibrutinib was the first BTK inhibitor to be approved by the FDA in 2015, with no hepatotoxicity reported before FDA approval. Since then, four case reports have highlighted its potential risk of severe liver injury and acute liver failure [14–17]. Furthermore, out of 36,732 ibrutinib-related reports made to the WHO, 509 were of hepatobiliary adverse reaction including 62 described as hepatic failure [18]. Moreover, the results of the phase 3 clinical trial comparing ibrutinib with zanubrutinib in patients with WM have recently been published. Following a median treatment duration of over 18 months, ibrutinib had to be stopped in 2% of patients due to hepatitis and drug-induced liver injury, whereas there was no report of liver injury in the zanubritinib arm. Acalabrutinib was the second BTK inhibitor to be approved in 2017. To date, there are no case reports of acalabrutinib-induced liver injury. However, there are three reports of hepatotoxicity in the WHO pharmacovigilance database but no reports of acute liver failure [18].

**Table 2:** Summary of case reports of other BTK inhibitors-induced liver injuries

Author title and date	Nandikolla, 2017 [14]	Kahn 2017 [15]	Kleijwegt 2019 [16]	Tafesh 2019 [17]
Underlying illness	Relapsing, refractory CLL	Refractory WM	Refractory WM	Refractory WM
BTK inhibitor	Ibrutinib	Ibrutinib	Ibrutinib	Ibrutinib
Patient’s age and gender	62-year-old male	59-year-old female	48-year-old female	77-year-old Female
Time of liver injury since starting BTK inhibitor	2 weeks	9 months	11 weeks	2 months
Pattern of DILI	Hepatocellular	Hepatocellular	Hepatocellular	Hepatocellular
Causality assessment/RUCAM	1 “Unlikely”^a^	7 “probable”	8 “probable”^a^	6 “possible”^a^
Liver histology	Centrilobular intrahepatic and canalicular cholestasis, ceroid-laden macrophages and minor apoptosis	Acute hepatitis with mixed acute and chronic inflammation and hepatocellular cholestasis	Not performed	Mixed inflammatory cell infiltrate, lobular disarray, hepatocellular ballooning, focal canalicular cholestasis, and necrosis
Intervention	Ibrutinib stopped	Ibrutinib stopped	Ibrutinib stopped	Ibrutinib stopped + oral prednisolone 60 mg daily for 12 days, then tapered down.
Outcome	Liver enzymes improved but did not normalise, and the patient died from the original illness.	Liver enzymes normalised after 60 days	Liver enzymes normalised after 5 weeks of stopping Ibrutinib	Liver enzymes normalised after 7 weeks and remained normal after 3 months

^a^RUCAM score was not reported; it was calculated based on the published data in the case report

As shown in Table 2, the indication for ibrutinib in three out of four case reports was refractory Waldenström’s macroglobulinaemia, as in our case. The age of patients ranged from 48 to 77, and the pattern of liver injury was hepatocellular in all cases, similar to the zanubrutinib case. The time of onset of DILI in our patient, 30 months, was significantly longer than reported cases of ibrutinib-induced DILI cases, ranging from 2 weeks to 9 months. The course of DILI, however, was similar to other cases, with liver biochemistry normalisation within 8 weeks after cessation of the drug. The RUCAM score was reported in one case as “probable” [15], and we calculated the updated RUCAM scoring using the available published data in the other case reports. The causality assessment for the first published case report of ibrutinib was “unlikely” due to an unexplained recurrent increase of liver enzymes and lack of hepatitis A serology [14]. In contrast, the other two cases were graded as “possible” [17] and “probable” [16].

The histopathology, in this case, was strongly corroborative of drug-induced aetiology, and the clinical context and temporal evolution provided a strong correlation. Other differentials such as idiopathic autoimmune hepatitis (AIH), large duct obstruction and infection were also considered but lacked clinical and serological correlations. The clinical context and the combined acute liver injury, with histological evidence of cholestasis with a mixed inflammatory infiltrate that included eosinophils, indicated causal association with drug aetiology. The liver biopsy may indeed be of help in cases, such as ours, to support the diagnosis of DILI. Previous studies comparing liver biopsies from patients with DILI and idiopathic AIH highlighted that the absence of fibrosis and the presence of cholestasis favour the diagnosis of DILI [19]. Although the definition of drug-induced AIH is not well-established, immune mechanisms may have contributed to this as the event resolved without immunosuppression. The mixed acute hepatitis picture with cholestasis was quite similar to that observed for ibrutinib by Kahn [15] and Tafesh [17]. A predominantly cholestatic injury was observed in the liver biopsy by Nandikolla [14] for ibrutinib, a histopathological pattern that may also be possible for the BTK group of drugs. Indeed, as the broader literature on DILI suggests, drugs/toxins may cause any pattern of liver injury, and it is important to meticulously record DILI patterns for new drugs with registries and biorepositories.

Zanubrutinib is metabolised in the liver via the cytochrome P450 system, largely by CYP 3A4, which is susceptible to drug-drug interactions with agents that inhibit or induce its activity. In this case, the patient was not exposed to any agent that could have interfered with the CYP enzymes. In contrast, it is important to note that significant inhibition of B cell activity from Bruton’s kinase inhibitors can cause viral replication and reactivation of hepatitis B, which may mimic drug-induced liver injury [20].

The pathogenesis of BTK inhibitor-induced liver toxicity remains unclear. Several mechanisms have been proposed, including immune mechanisms due to genetic variants, oxidative damage, and direct hepatoxicity via mitochondrial dysfunction [21]. Metabolic bioactivation of small molecule kinase inhibitors by cytochrome P450 enzymes and generating chemically reactive products is presumed to be the initiating event in tyrosine kinase inhibitor-induced hepatotoxicity [22]. Recent in vitro mechanistic studies investigated potential cellular pathways of tyrosine kinase inhibitors-induced hepatotoxicity by studying their effect on mitochondrial function using hepatic cell lines, HepG2 and HepaRG cells [23, 24]. It is worth noting that HepG2 cells lack cytochrome P450 drug activity, which poses a significant limitation to investigate the role of pathways of biotransformation [22]. Further mechanistic studies investigating cellular signalling and pathways of BTK inhibitors are needed to gain a better understanding of the pathogenesis of BTK inhibitor-induced liver injury.

In conclusion, physicians should be aware of the potential liver toxicity of zanubrutinib, which requires vigilance. When suspecting liver injury, zanubrutinib should be withheld instantly, and reactivation of hepatitis B must be excluded as a potential cause.

## Data Availability

All data generated or analysed during this study are included in this published article.

## Abbreviations

AIH: Autoimmune hepatitis
ALP: Alkaline phosphatase
ALT: Alanine aminotransferase
Alb: Albumin
AMA: Antimitochondrial antibodies
ANA: Antinuclear antibodies
anti-CMV: Cytomegalovirus antibody
anti-EBV: Anti-Epstein-Barr virus antibody
anti-HAV: Hepatitis A virus antibody
anti-HCV: Hepatitis C virus antibody
anti-HEV: Hepatitis E virus antibody
APTT: Activated partial thromboplastin time
ASMA: Anti Smooth Muscle antibodies
AST: Aspartate aminotransferase
BMI: Body mass index
BTK: Bruton’s tyrosine kinase
CRP: C-reactive protein
DILI: Drug-induced liver injury
HBsAg: Hepatitis B surface antigen
IgA: Immunoglobulin A
IgG: Immunoglobulin G
IgM: Immunoglobulin M
LKM-1: Liver Kidney Microsomal antibody
PT: Prothrombin time
RUCAM: Roussel Uclaf Causality Assessment Method
TB: Total bilirubin
ULN: Upper limits of normal
WM: Waldenström’s macroglobulinaemia
